# The Nursing of Rural Fever Patients

**Published:** 1920-01-31

**Authors:** 


### THE MATRONS' AND SISTERS' DEPARTMENT.

### THE NURSING OF RURAL FEVER PATIENTS.

AMONG the anomalies in our national system of nursing none are more peculiar than those which attend the care of the fever-stricken in the country. Largely owing to the energy of the Fever Nurses' Association, founded by the late Dr. Biernacki, the training of fever nurses has, during recent years, become highly standardised; duly qualified fever nurses are now perhaps more efficiently prepared for their work than any other class of special nurses. They have fully established their claim to a separate register among trained nurss.

Is special training indispensable for women who undertake the care of the fever-stricken? This is a question which scarcely requires an answer, although women trained in general hospitals are often called on to undertake fever patients in private practice. The public is probably under the impression that skilled fever nursing, with supervision and attention night and day, is provided in all fever institutions under public control. In other less happily organised countries people may be content to learn that fever patients are merely huddled out of the way without much attempt to relieve their sufferings. But in Great Britain all is done always on the best possible lines. Fortunate, indeed, are they whom an enlightened system of public health removes from their homes to be tended under municipal control.

The plain fact is that the little isolation hospitals scattered up and down throughout the country are under no system of public inspection and are exposed to some of the worst abuses which result from parsimony and neglect. They are usually a mile or two from a town, and away from other houses. They are probably empty for part of the year, though at any time a sudden call for beds may tax their resources to the utmost. The aim of the managers is to pay out as little as possible in wages or salaries, and a married couple, the wife possessing some nursing experience, is the desideratum. This nurse, or caretaker, is expected to deal single-handed with any patients up to a certain number who may present themselves, and the old impression that fevers are "childish complaints," and that patients need only warmth and regular meals, will be found flourishing among the managers. The upkeep of these little establishments is frequently deplorable. No one ever visits them except a hurried M.O.H., who is far, indeed, from desiring to inspect the linen cupboard or overhaul the general equipment. The caretakers speedily learn that requests for repairs and renewals are not received with particular favour by managers mainly exercised to keep down expenses, and supplementary labour is often hard to get owing to public dread of infection.

But it is the nursing which patients get under these conditions to which we desire to draw our readers' attention. When the nurse-caretaker has the whole work of the house on her hands in addition to the care of three or four patients, what attention can she give them? In one instance which came under the writer's notice seven patients, suffering respectively from diphtheria and scarlet fever, were allowed to accumulate before the services of another nurse were procured. It goes without saying that patients under such conditions have to be left severely alone at night. No woman single-handed could carry on through the entire day and give even the most perfunctory attention to the wards at night. Ought any young sick children, ought any fever patients to be left shut in for twelve hours during the night without attendance?

It is the sharp contrast between theory and practice to which we would draw attention. On the one hand, for townsfolk, exact precautions, a high degree of technical skill in the nurses, continuous care. On the other hand, for country-folk, neglect of the most elementary precautions, and something so like cruelty to the sick as to be hardly distinguishable save in intention.

That there are difficulties in the organisation of small isolation hospitals is as obvious as that these difficulties can be overcome. The first need is for inspection and control. The bodies by which they are ruled are often ill-suited for the task. They need to be reinforced by guidance from headquarters in such matters as a minimum nursing staff, a proper equipment, a dietary for the sick. There is need especially for an adequate service of supply fever nurses ready to take up duty in any locality without delay. Among the tasks which lie ready to the hand of the Ministry of Health none is more urgent than this.

